# Methyl 5′-(2-hy­droxy­phen­yl)-4′,5′,6′,7′-tetra­hydro­spiro­[2*H*-1-benzopyran-2,7′-1,2,4-triazolo[1,5-*a*]pyrimidine]-3-carboxyl­ate

**DOI:** 10.1107/S160053681005052X

**Published:** 2010-12-11

**Authors:** Viktor Kettmann, Jan Světlík

**Affiliations:** a Faculty of Pharmacy, Comenius University, Odbojarov 10, SK-83232 Bratislava, Slovakia

## Abstract

There are two crystallographically independent mol­ecules in the asymmetric unit of the title compound, C_21_H_18_N_4_O_4_. The substituted benzopyran portion of one of the independent mol­ecules exhibits disorder [occupancy 0.5248 (18):0.4752 (18)], which was modelled by using two sets of atomic positions and restraints on the chemically equivalent bond lengths and angles. The central, partially saturated pyrimidine rings of both independent mol­ecules were found to assume unsymmetrical half-chair conformations. The hy­droxy­phenyl substituent occupies an equatorial position in both mol­ecules, and is rotated by 55.6 (1)° from the mean plane of the pyrimidine ring in one independent mol­ecule, and by 53.4 (1)° in the other. In the crystal, there are two types of inter­molecular hydrogen bond present: reciprocal N—H⋯N inter­actions join the two crystallographically independent mol­ecules into a dimer and O—H⋯N inter­actions link the dimers into sheets in the *ab* plane.

## Related literature

For our work on the preparation of novel pyrazolo­pyridines and oxygen-bridged pyrazolo-, tetra­zolo-, benzimidazo- and thia­zolopyrimidines, see: Světlík *et al.* (2010 ▶). For the synthesis of the title compound, see: Světlík & Kettmann (2011 ▶). For biological aspects of Biginelli compounds in general, see: Kappe (2000 ▶).
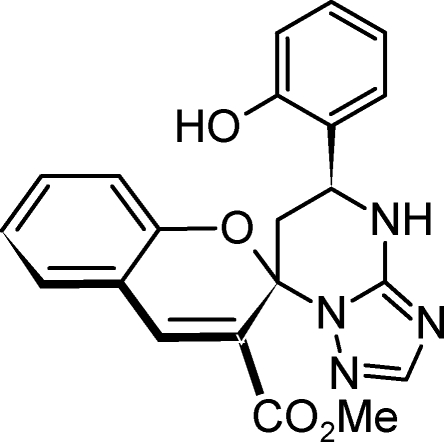


## Experimental

### 

#### Crystal data

C_21_H_18_N_4_O_4_
*M* *_r_* = 390.39
Monoclinic, 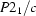

*a* = 19.337 (6) Å
*b* = 14.824 (5) Å
*c* = 13.932 (5) Å
β = 97.94 (1)°
*V* = 3955 (2) Å^3^
*Z* = 8
Mo *K*α radiation
μ = 0.09 mm^−1^
*T* = 296 K
0.30 × 0.25 × 0.20 mm

#### Data collection

Siemens P4 diffractometer
12311 measured reflections
10237 independent reflections
5854 reflections with *I* > 2σ(*I*)
*R*_int_ = 0.050
3 standard reflections every 97 reflections  intensity decay: none

#### Refinement

*R*[*F* ^2^ > 2σ(*F* ^2^)] = 0.053
*wR*(*F* ^2^) = 0.134
*S* = 1.01
10237 reflections
645 parameters
90 restraints
H-atom parameters constrained
Δρ_max_ = 0.16 e Å^−3^
Δρ_min_ = −0.20 e Å^−3^

### 

Data collection: *XSCANS* (Siemens, 1994 ▶); cell refinement: *XSCANS*; data reduction: *XSCANS*; program(s) used to solve structure: *SHELXS97* (Sheldrick, 2008 ▶); program(s) used to refine structure: *SHELXL97* (Sheldrick, 2008 ▶); molecular graphics: *PLATON* (Spek, 2009 ▶); software used to prepare material for publication: *SHELXL97*.

## Supplementary Material

Crystal structure: contains datablocks global, I. DOI: 10.1107/S160053681005052X/nk2069sup1.cif

Structure factors: contains datablocks I. DOI: 10.1107/S160053681005052X/nk2069Isup2.hkl

Additional supplementary materials:  crystallographic information; 3D view; checkCIF report

## Figures and Tables

**Table 1:** Hydrogen-bond geometry (Å, °)

*D*—H⋯*A*	*D*—H	H⋯*A*	*D*⋯*A*	*D*—H⋯*A*
N4—H4⋯N7	0.86	2.21	3.0156 (19)	156
O4—H4*C*⋯N2^i^	0.82	2.12	2.9152 (17)	163
N8—H8*A*⋯N3	0.86	2.10	2.9168 (19)	159
O8—H8*B*⋯N6^ii^	0.82	2.09	2.8898 (19)	164

Symmetry codes: (i) 
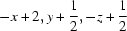
; (ii) 
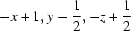
.

**Fig. 1.**
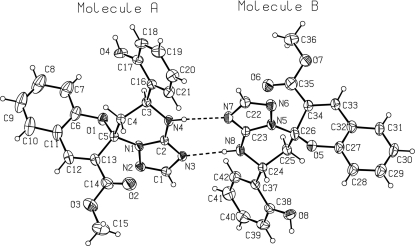
Displacement ellipsoid plot of the two independent molecules of (I) with the labelling scheme for the non-H atoms, which are drawn as 35% probability ellipsoids. The minor disorder component of molecule B is omitted for clarity. Hydrogen bonds are illustrated by dashed lines.

## supplementary crystallographic information

### Comment

In recent years, products of the Biginelli-like reaction have attracted much attention due to their significant pharmacological activities (Kappe, 2000). Our recent contribution to this subject concerns preparation of novel pyrazolopyridines and oxygen-bridged pyrazolo-, tetrazolo-, benzimidazo- and thiazolopyrimidines (Světlík *et al.*, 2010). Quite recently, we were interested in the behaviour of 3-amino-1,2,4-triazole in related cyclizations while varying the structure of the aldehydic component (Světlík & Kettmann, 2011). As the structure of the product from the cyclocondensation of salicylaldehyde, methyl acetoacetate and the above amine was more complex, we decided to verify the most probable structure (I) (based on NMR) by using a single-crystal X-ray analysis. Another aim of this work was to obtain complete structural data indispensable for studying structure-activity relationships.

The structure determination (Fig.1) confirmed the spectroscopic assignments, *i.e.* (I) is indeed the correct structure of the compound. The asymmetric unit of the structure comprises two molecules (A and B) of (I). Moreover, the methoxycarbonylbenzopyran moiety of molecule B is disordered between two well defined positions (denoted by unprimed and primed atomic symbols) with approximately equal occupancies. The disordered sites differ in (i) *ca* 20°-rotation of the substituted benzopyran ring about an axis passing through the spiro (C26) atom and perpendicular to the mean plane of the ring and (ii) the conformation of the ester group.

As mentioned above, from the pharmacological viewpoint the most important aspect of the molecular structure (Fig.1) concerns three-dimensional disposition of the key functional (pharmacophoric) elements (hydrophobic groups and heteroatoms able to form hydrogen bonds) which can be expressed in terms of the conformationl parameters of the molecule. Thus, the conformation of the central pyrimidine ring in both molecules can best be described as an unsymmetrical half-chair. The hydroxyphenyl ring occupies an equatorial position on C3 and is inclined at an angle of 55.6 (1)° to the mean plane of the heterocycle. The corresponding dihedral angle in molecule B is 53.4 (1)°. As to the conformation of the ester group, its carbonyl bond is oriented *anti* (with respect to the neighbouring double bond of the benzopyran moiety) in molecule A and the unprimed sites of molecule B while the orientation is *syn* for the primed isomer.

In the crystal, there are two types of intermolecular hydrogen bond present: reciprocal N–H···N interactions join the two crystallographically independent molecules into a dimer (Figure 1) and an O–H···N interaction links the dimers into a two-dimensional sheet in the *ab* plane.

### Experimental

Synthesis of the title compound, (I), is described elsewhere (Světlík & Kettmann, 2011). In short, to a solution of 3-amino-1,2,4-triazole (0.35 g, 4.0 mmol) in ethanol (20 ml) were added salicylaldehyde (8.0 mmol) and methyl acetoacetate (0.44 ml, 4.0 mmol). The mixture contained 4 drops of concentrated HCl and was refluxed for 20 h. After cooling the solution was left to crystallize. The crude product was finally recrystallized from acetonitrile (51% yield; m.p. 511–512 K).

### Refinement

H atoms were treated as riding atoms with distances C—H = 0.93 Å (CH_arom_), 0.97 Å (CH_2_), 0.98 Å (CH), 0.96 Å (CH_3_), N—H = 0.86 Å and O—H = 0.82 Å; *U*_iso_ of the H atoms were set to 1.2 (1.5 for the methyl H atoms) times *U*_eq_ of the parent atom. The disorder of the methoxycarbonylbenzopyran fragment of molecule B was modelled by resolving the atomic positions into two components and using a total of 90 restraints on equivalent bond distances and angles [use of the SAME instruction in *SHELXL97* (Sheldrick, 2008)]. This resulted in a model with (refined) occupancies of the unprimed and primed sites of 47.5 (3) and 52.5 (3)%, respectively, and a reasonable geometry for both isomers.

### Crystal data

**Table:**

C_21_H_18_N_4_O_4_	*F*(000) = 1632
*M**_r_* = 390.39	*D*_x_ = 1.311 Mg m^−^^3^
Monoclinic, *P*2_1_/*c*	Melting point: 511 K
Hall symbol: -P 2ybc	Mo *K*α radiation, λ = 0.71073 Å
*a* = 19.337 (6) Å	Cell parameters from 20 reflections
*b* = 14.824 (5) Å	θ = 7–18°
*c* = 13.932 (5) Å	µ = 0.09 mm^−^^1^
β = 97.94 (1)°	*T* = 296 K
*V* = 3955 (2) Å^3^	Prism, colourless
*Z* = 8	0.30 × 0.25 × 0.20 mm

### Data collection

**Table:**

Siemens P4 diffractometer	*R*_int_ = 0.050
Radiation source: fine-focus sealed tube	θ_max_ = 28.8°, θ_min_ = 1.1°
graphite	*h* = −26→26
ω/2θ scans	*k* = −20→1
12311 measured reflections	*l* = −1→18
10237 independent reflections	3 standard reflections every 97 reflections
5854 reflections with *I* > 2σ(*I*)	intensity decay: none

### Refinement

**Table:**

Refinement on *F*^2^	Primary atom site location: structure-invariant direct methods
Least-squares matrix: full	Secondary atom site location: difference Fourier map
*R*[*F*^2^ > 2σ(*F*^2^)] = 0.053	Hydrogen site location: inferred from neighbouring sites
*wR*(*F*^2^) = 0.134	H-atom parameters constrained
*S* = 1.01	*w* = 1/[σ^2^(*F*_o_^2^) + (0.0583*P*)^2^ + 0.3383*P*] where *P* = (*F*_o_^2^ + 2*F*_c_^2^)/3
10237 reflections	(Δ/σ)_max_ = 0.001
645 parameters	Δρ_max_ = 0.16 e Å^−^^3^
90 restraints	Δρ_min_ = −0.20 e Å^−^^3^

### Special details

**Table:**

Geometry. All e.s.d.'s (except the e.s.d. in the dihedral angle between two l.s. planes) are estimated using the full covariance matrix. The cell e.s.d.'s are taken into account individually in the estimation of e.s.d.'s in distances, angles and torsion angles; correlations between e.s.d.'s in cell parameters are only used when they are defined by crystal symmetry. An approximate (isotropic) treatment of cell e.s.d.'s is used for estimating e.s.d.'s involving l.s. planes.
Refinement. Refinement of *F*^2^ against ALL reflections. The weighted *R*-factor *wR* and goodness of fit *S* are based on *F*^2^, conventional *R*-factors *R* are based on *F*, with *F* set to zero for negative *F*^2^. The threshold expression of *F*^2^ > σ(*F*^2^) is used only for calculating *R*-factors(gt) *etc*. and is not relevant to the choice of reflections for refinement. *R*-factors based on *F*^2^ are statistically about twice as large as those based on *F*, and *R*- factors based on ALL data will be even larger.

### Fractional atomic coordinates and isotropic or equivalent isotropic displacement parameters (Å^2^)

**Table:**

	*x*	*y*	*z*	*U*_iso_*/*U*_eq_	Occ. (<1)
N1	0.89134 (6)	0.49569 (8)	0.23684 (9)	0.0380 (3)	
N2	0.87417 (6)	0.41939 (8)	0.28645 (9)	0.0413 (3)	
C1	0.81119 (8)	0.43874 (10)	0.30354 (11)	0.0423 (4)	
H1	0.7857	0.3993	0.3369	0.051*	
N3	0.78455 (6)	0.52028 (8)	0.26983 (10)	0.0413 (3)	
C2	0.83683 (7)	0.55417 (10)	0.22834 (10)	0.0362 (3)	
N4	0.84032 (6)	0.63458 (8)	0.18381 (10)	0.0430 (3)	
H4	0.8037	0.6669	0.1671	0.052*	
C3	0.90931 (8)	0.66378 (10)	0.16555 (11)	0.0394 (3)	
H3	0.9373	0.6789	0.2275	0.047*	
C4	0.94364 (8)	0.58371 (10)	0.12003 (11)	0.0403 (3)	
H4A	0.9154	0.5672	0.0596	0.048*	
H4B	0.9892	0.6018	0.1054	0.048*	
C5	0.95183 (8)	0.50135 (10)	0.18751 (11)	0.0402 (3)	
O1	1.00884 (5)	0.51615 (8)	0.26455 (8)	0.0508 (3)	
C6	1.07550 (9)	0.49704 (15)	0.24576 (13)	0.0616 (5)	
C7	1.12993 (10)	0.5448 (2)	0.29503 (16)	0.0905 (8)	
H7	1.1217	0.5906	0.3378	0.109*	
C8	1.19812 (12)	0.5235 (3)	0.27976 (19)	0.1107 (10)	
H8	1.2356	0.5557	0.3121	0.133*	
C9	1.20987 (12)	0.4559 (3)	0.2179 (2)	0.1105 (10)	
H9	1.2555	0.4421	0.2091	0.133*	
C10	1.15617 (11)	0.4082 (2)	0.16873 (17)	0.0901 (8)	
H10	1.1654	0.3626	0.1265	0.108*	
C11	1.08687 (10)	0.42749 (15)	0.18141 (14)	0.0654 (5)	
C12	1.02669 (10)	0.38182 (13)	0.13138 (13)	0.0586 (5)	
H12	1.0326	0.3292	0.0971	0.070*	
C13	0.96249 (9)	0.41406 (11)	0.13359 (12)	0.0492 (4)	
C14	0.89967 (11)	0.37507 (14)	0.07541 (14)	0.0644 (5)	
O2	0.84434 (8)	0.41265 (11)	0.05622 (11)	0.0803 (4)	
O3	0.91120 (9)	0.28860 (11)	0.04917 (13)	0.0974 (5)	
C15	0.85174 (15)	0.2483 (2)	−0.0140 (2)	0.1287 (12)	
H15A	0.8114	0.2478	0.0193	0.193*	
H15B	0.8632	0.1876	−0.0300	0.193*	
H15C	0.8419	0.2832	−0.0723	0.193*	
C16	0.90575 (8)	0.74627 (10)	0.09945 (11)	0.0411 (4)	
C17	0.96473 (9)	0.80048 (10)	0.10207 (12)	0.0447 (4)	
C18	0.96418 (10)	0.87454 (12)	0.04036 (13)	0.0570 (5)	
H18	1.0034	0.9113	0.0431	0.068*	
C19	0.90558 (11)	0.89336 (13)	−0.02473 (14)	0.0644 (5)	
H19	0.9059	0.9420	−0.0668	0.077*	
C20	0.84611 (10)	0.84041 (13)	−0.02812 (14)	0.0625 (5)	
H20	0.8065	0.8536	−0.0716	0.075*	
C21	0.84671 (9)	0.76719 (12)	0.03459 (13)	0.0533 (4)	
H21	0.8069	0.7317	0.0331	0.064*	
O4	1.02225 (6)	0.77752 (8)	0.16686 (9)	0.0573 (3)	
H4C	1.0519	0.8172	0.1678	0.086*	
N5	0.60625 (7)	0.76363 (8)	0.24160 (10)	0.0455 (3)	
N6	0.61876 (7)	0.83878 (9)	0.18647 (11)	0.0535 (4)	
C22	0.67762 (9)	0.81715 (12)	0.15613 (14)	0.0556 (5)	
H22	0.6994	0.8551	0.1164	0.067*	
N7	0.70520 (7)	0.73559 (9)	0.18622 (12)	0.0558 (4)	
C23	0.65830 (8)	0.70369 (11)	0.23847 (13)	0.0465 (4)	
N8	0.65829 (7)	0.62390 (9)	0.28501 (11)	0.0540 (4)	
H8A	0.6955	0.5916	0.2964	0.065*	
C24	0.59260 (8)	0.59469 (10)	0.31523 (12)	0.0430 (4)	
H24	0.5601	0.5791	0.2573	0.052*	
C25	0.56262 (9)	0.67436 (10)	0.36544 (13)	0.0490 (4)	
H25A	0.5947	0.6914	0.4223	0.059*	
H25B	0.5188	0.6569	0.3865	0.059*	
C26	0.55051 (10)	0.75491 (12)	0.29647 (15)	0.0603 (5)	
O5	0.48981 (18)	0.7585 (2)	0.2439 (3)	0.0514 (8)	0.4752 (18)
C27	0.4300 (3)	0.7844 (4)	0.2838 (5)	0.0515 (14)	0.4752 (18)
C28	0.3677 (2)	0.7475 (4)	0.2439 (4)	0.0598 (12)	0.4752 (18)
H28	0.3655	0.7049	0.1946	0.072*	0.4752 (18)
C29	0.3078 (6)	0.7763 (12)	0.2797 (15)	0.068 (3)	0.4752 (18)
H29	0.2648	0.7520	0.2542	0.081*	0.4752 (18)
C30	0.31096 (19)	0.8398 (3)	0.3519 (3)	0.0635 (12)	0.4752 (18)
H30	0.2703	0.8587	0.3746	0.076*	0.4752 (18)
C31	0.37280 (18)	0.8745 (3)	0.3897 (3)	0.0590 (10)	0.4752 (18)
H31	0.3743	0.9181	0.4379	0.071*	0.4752 (18)
C32	0.4350 (4)	0.8469 (7)	0.3585 (6)	0.0531 (18)	0.4752 (18)
C33	0.50333 (18)	0.8820 (3)	0.3960 (3)	0.0523 (9)	0.4752 (18)
H33	0.5073	0.9313	0.4377	0.063*	0.4752 (18)
C34	0.5608 (2)	0.8434 (3)	0.3703 (4)	0.0513 (10)	0.4752 (18)
C35	0.6308 (7)	0.8748 (16)	0.409 (3)	0.075 (7)	0.4752 (18)
O6	0.68391 (16)	0.8315 (2)	0.4169 (2)	0.0679 (8)	0.4752 (18)
O7	0.63054 (16)	0.9545 (2)	0.4629 (2)	0.0733 (9)	0.4752 (18)
C36	0.6950 (2)	0.9830 (4)	0.5187 (4)	0.0913 (17)	0.4752 (18)
H36A	0.7315	0.9823	0.4785	0.137*	0.4752 (18)
H36B	0.7068	0.9426	0.5724	0.137*	0.4752 (18)
H36C	0.6897	1.0430	0.5425	0.137*	0.4752 (18)
O5'	0.49251 (17)	0.7216 (2)	0.2109 (3)	0.0570 (8)	0.5248 (18)
C27'	0.42271 (17)	0.7236 (2)	0.2215 (3)	0.0531 (9)	0.5248 (18)
C28'	0.37876 (18)	0.6636 (3)	0.1708 (3)	0.0663 (10)	0.5248 (18)
H28'	0.3957	0.6209	0.1312	0.080*	0.5248 (18)
C29'	0.3080 (2)	0.6672 (3)	0.1793 (3)	0.0753 (12)	0.5248 (18)
H29'	0.2775	0.6261	0.1454	0.090*	0.5248 (18)
C30'	0.2826 (2)	0.7305 (3)	0.2368 (4)	0.0707 (12)	0.5248 (18)
H30'	0.2354	0.7309	0.2435	0.085*	0.5248 (18)
C31'	0.3258 (5)	0.7922 (11)	0.2837 (14)	0.065 (3)	0.5248 (18)
H31'	0.3080	0.8367	0.3206	0.079*	0.5248 (18)
C32'	0.3977 (3)	0.7896 (4)	0.2773 (4)	0.0529 (12)	0.5248 (18)
C33'	0.4482 (4)	0.8512 (6)	0.3314 (6)	0.0536 (17)	0.5248 (18)
H33'	0.4324	0.9002	0.3638	0.064*	0.5248 (18)
C34'	0.5176 (2)	0.8370 (2)	0.3345 (3)	0.0510 (9)	0.5248 (18)
C35'	0.5655 (2)	0.8960 (3)	0.3948 (3)	0.0578 (10)	0.5248 (18)
O6'	0.55115 (14)	0.96768 (19)	0.4274 (2)	0.0794 (9)	0.5248 (18)
O7'	0.6326 (4)	0.8630 (9)	0.4186 (13)	0.056 (2)	0.5248 (18)
C36'	0.6826 (2)	0.9153 (3)	0.4829 (4)	0.0819 (13)	0.5248 (18)
H36D	0.6833	0.9762	0.4596	0.123*	0.5248 (18)
H36E	0.7282	0.8891	0.4849	0.123*	0.5248 (18)
H36F	0.6694	0.9152	0.5468	0.123*	0.5248 (18)
C37	0.60185 (8)	0.51266 (10)	0.38106 (12)	0.0428 (4)	
C38	0.54356 (9)	0.45826 (11)	0.38742 (12)	0.0469 (4)	
C39	0.55036 (10)	0.38129 (12)	0.44557 (13)	0.0575 (5)	
H39	0.5119	0.3445	0.4494	0.069*	
C40	0.61396 (12)	0.36000 (14)	0.49714 (14)	0.0676 (5)	
H40	0.6180	0.3089	0.5362	0.081*	
C41	0.67168 (11)	0.41263 (14)	0.49217 (15)	0.0685 (5)	
H41	0.7147	0.3969	0.5266	0.082*	
C42	0.66511 (9)	0.48984 (12)	0.43498 (13)	0.0561 (4)	
H42	0.7038	0.5268	0.4329	0.067*	
O8	0.48171 (6)	0.48419 (8)	0.33605 (9)	0.0604 (3)	
H8B	0.4576	0.4395	0.3211	0.091*	

### Atomic displacement parameters (Å^2^)

**Table:**

	*U*^11^	*U*^22^	*U*^33^	*U*^12^	*U*^13^	*U*^23^
N1	0.0396 (7)	0.0328 (6)	0.0429 (7)	0.0076 (5)	0.0102 (5)	0.0032 (5)
N2	0.0468 (7)	0.0324 (7)	0.0455 (7)	0.0047 (5)	0.0086 (6)	0.0056 (6)
C1	0.0433 (9)	0.0342 (8)	0.0501 (9)	−0.0004 (7)	0.0092 (7)	0.0036 (7)
N3	0.0371 (7)	0.0351 (7)	0.0530 (8)	0.0039 (5)	0.0111 (6)	0.0048 (6)
C2	0.0329 (7)	0.0349 (8)	0.0413 (8)	0.0050 (6)	0.0066 (6)	−0.0001 (6)
N4	0.0377 (7)	0.0358 (7)	0.0571 (8)	0.0074 (5)	0.0123 (6)	0.0100 (6)
C3	0.0385 (8)	0.0373 (8)	0.0436 (8)	0.0020 (6)	0.0103 (7)	0.0042 (7)
C4	0.0372 (8)	0.0425 (8)	0.0423 (8)	0.0055 (7)	0.0098 (7)	0.0050 (7)
C5	0.0351 (8)	0.0438 (9)	0.0419 (8)	0.0063 (6)	0.0060 (6)	−0.0004 (7)
O1	0.0399 (6)	0.0646 (8)	0.0467 (6)	0.0063 (5)	0.0021 (5)	−0.0030 (6)
C6	0.0411 (10)	0.0908 (15)	0.0516 (10)	0.0061 (9)	0.0013 (8)	0.0124 (10)
C7	0.0531 (13)	0.145 (2)	0.0712 (14)	−0.0062 (14)	−0.0014 (11)	−0.0063 (15)
C8	0.0517 (14)	0.197 (3)	0.0796 (17)	−0.0094 (17)	−0.0031 (12)	−0.006 (2)
C9	0.0477 (13)	0.198 (3)	0.0851 (18)	0.0209 (17)	0.0068 (13)	0.011 (2)
C10	0.0572 (13)	0.139 (2)	0.0760 (15)	0.0342 (14)	0.0150 (12)	0.0160 (15)
C11	0.0517 (11)	0.0894 (15)	0.0566 (11)	0.0265 (11)	0.0124 (9)	0.0150 (11)
C12	0.0631 (12)	0.0615 (11)	0.0540 (10)	0.0215 (9)	0.0186 (9)	0.0037 (9)
C13	0.0525 (10)	0.0483 (10)	0.0492 (10)	0.0102 (8)	0.0147 (8)	0.0012 (8)
C14	0.0759 (14)	0.0581 (12)	0.0602 (12)	−0.0013 (11)	0.0132 (10)	−0.0172 (10)
O2	0.0715 (10)	0.0787 (10)	0.0844 (10)	0.0002 (8)	−0.0118 (8)	−0.0185 (8)
O3	0.1045 (12)	0.0776 (11)	0.1106 (13)	−0.0004 (9)	0.0168 (10)	−0.0445 (10)
C15	0.125 (2)	0.109 (2)	0.150 (3)	−0.0275 (18)	0.010 (2)	−0.076 (2)
C16	0.0443 (9)	0.0376 (8)	0.0429 (8)	0.0013 (7)	0.0115 (7)	0.0029 (7)
C17	0.0519 (10)	0.0372 (8)	0.0454 (9)	−0.0023 (7)	0.0081 (7)	0.0010 (7)
C18	0.0695 (12)	0.0413 (9)	0.0606 (11)	−0.0124 (8)	0.0102 (9)	0.0083 (8)
C19	0.0812 (14)	0.0485 (10)	0.0623 (12)	−0.0019 (10)	0.0056 (10)	0.0197 (9)
C20	0.0663 (12)	0.0570 (11)	0.0618 (12)	0.0059 (9)	0.0001 (9)	0.0176 (10)
C21	0.0468 (9)	0.0547 (10)	0.0579 (11)	0.0014 (8)	0.0061 (8)	0.0130 (9)
O4	0.0587 (8)	0.0490 (7)	0.0604 (8)	−0.0162 (6)	−0.0047 (6)	0.0063 (6)
N5	0.0439 (7)	0.0350 (7)	0.0607 (9)	0.0095 (6)	0.0181 (6)	0.0077 (6)
N6	0.0576 (9)	0.0399 (8)	0.0666 (9)	0.0099 (6)	0.0212 (7)	0.0152 (7)
C22	0.0550 (11)	0.0438 (10)	0.0725 (12)	0.0037 (8)	0.0249 (9)	0.0136 (9)
N7	0.0452 (8)	0.0437 (8)	0.0835 (11)	0.0089 (6)	0.0273 (7)	0.0165 (8)
C23	0.0387 (8)	0.0379 (8)	0.0662 (11)	0.0050 (7)	0.0184 (8)	0.0050 (8)
N8	0.0431 (8)	0.0401 (7)	0.0845 (11)	0.0124 (6)	0.0287 (7)	0.0161 (7)
C24	0.0399 (8)	0.0363 (8)	0.0554 (10)	0.0022 (6)	0.0158 (7)	0.0026 (7)
C25	0.0506 (10)	0.0409 (9)	0.0593 (10)	0.0082 (7)	0.0215 (8)	0.0052 (8)
C26	0.0529 (11)	0.0502 (11)	0.0834 (14)	0.0186 (8)	0.0295 (10)	0.0149 (10)
O5	0.0439 (17)	0.053 (2)	0.059 (2)	0.0014 (16)	0.0131 (14)	−0.0048 (15)
C27	0.040 (3)	0.049 (3)	0.065 (3)	0.011 (3)	0.008 (3)	0.014 (2)
C28	0.045 (3)	0.065 (3)	0.069 (3)	0.006 (2)	0.003 (2)	0.012 (2)
C29	0.049 (5)	0.070 (9)	0.083 (6)	0.007 (5)	0.008 (5)	0.018 (5)
C30	0.035 (2)	0.075 (3)	0.082 (3)	0.0120 (19)	0.013 (2)	0.014 (2)
C31	0.045 (2)	0.061 (2)	0.074 (3)	0.0140 (18)	0.0163 (19)	0.007 (2)
C32	0.046 (3)	0.053 (3)	0.061 (4)	0.012 (3)	0.010 (3)	0.010 (3)
C33	0.044 (2)	0.047 (2)	0.067 (2)	0.0093 (17)	0.0108 (19)	0.0010 (19)
C34	0.041 (2)	0.046 (3)	0.068 (3)	0.006 (2)	0.011 (2)	0.001 (2)
C35	0.066 (9)	0.058 (6)	0.099 (13)	0.008 (4)	0.008 (5)	0.012 (5)
O6	0.0509 (18)	0.0701 (19)	0.081 (2)	0.0059 (14)	0.0015 (15)	−0.0098 (16)
O7	0.0571 (19)	0.066 (2)	0.093 (2)	0.0034 (15)	−0.0009 (16)	−0.0188 (17)
C36	0.075 (3)	0.086 (4)	0.107 (4)	−0.014 (3)	−0.008 (3)	−0.038 (3)
O5'	0.0451 (15)	0.054 (2)	0.074 (2)	0.0078 (14)	0.0174 (15)	−0.0004 (15)
C27'	0.044 (2)	0.049 (2)	0.067 (2)	0.0067 (16)	0.0115 (17)	0.0111 (19)
C28'	0.055 (2)	0.056 (2)	0.088 (3)	0.0039 (17)	0.008 (2)	0.004 (2)
C29'	0.060 (2)	0.066 (3)	0.098 (3)	−0.005 (2)	0.006 (2)	0.005 (2)
C30'	0.054 (3)	0.066 (3)	0.092 (4)	0.003 (2)	0.009 (2)	0.010 (3)
C31'	0.058 (5)	0.053 (5)	0.086 (5)	0.009 (4)	0.011 (5)	0.015 (3)
C32'	0.040 (3)	0.051 (3)	0.068 (3)	0.007 (3)	0.010 (3)	0.011 (2)
C33'	0.054 (3)	0.043 (3)	0.066 (4)	0.018 (2)	0.014 (3)	0.007 (3)
C34'	0.046 (2)	0.0434 (19)	0.065 (2)	0.0124 (17)	0.0159 (19)	0.0037 (17)
C35'	0.052 (3)	0.052 (2)	0.071 (3)	0.007 (2)	0.0130 (19)	0.000 (2)
O6'	0.0669 (18)	0.0638 (17)	0.106 (2)	0.0106 (14)	0.0083 (15)	−0.0217 (16)
O7'	0.049 (4)	0.055 (4)	0.064 (3)	0.008 (2)	0.004 (2)	−0.004 (5)
C36'	0.067 (3)	0.078 (3)	0.096 (3)	−0.005 (3)	−0.001 (2)	−0.012 (3)
C37	0.0448 (9)	0.0348 (8)	0.0511 (9)	0.0028 (7)	0.0149 (7)	0.0005 (7)
C38	0.0538 (10)	0.0410 (9)	0.0485 (9)	−0.0034 (7)	0.0162 (8)	−0.0041 (7)
C39	0.0758 (13)	0.0452 (10)	0.0545 (11)	−0.0119 (9)	0.0197 (10)	0.0040 (8)
C40	0.0940 (16)	0.0537 (11)	0.0563 (12)	−0.0004 (11)	0.0146 (11)	0.0151 (9)
C41	0.0742 (13)	0.0662 (13)	0.0634 (12)	0.0109 (11)	0.0032 (10)	0.0129 (10)
C42	0.0541 (10)	0.0535 (10)	0.0613 (11)	−0.0003 (8)	0.0094 (9)	0.0067 (9)
O8	0.0501 (7)	0.0514 (7)	0.0788 (9)	−0.0130 (6)	0.0049 (6)	0.0032 (6)

### Geometric parameters (Å, °)

**Table:**

N1—C2	1.3573 (18)	C24—H24	0.9800
N1—N2	1.3895 (17)	C25—C26	1.530 (2)
N1—C5	1.4385 (19)	C25—H25A	0.9700
N2—C1	1.3046 (19)	C25—H25B	0.9700
C1—N3	1.3713 (19)	C26—O5	1.296 (4)
C1—H1	0.9300	C26—C34'	1.503 (4)
N3—C2	1.3303 (18)	C26—O5'	1.598 (4)
C2—N4	1.3498 (19)	C26—C34	1.663 (6)
N4—C3	1.4580 (19)	O5—C27	1.405 (6)
N4—H4	0.8600	C27—C28	1.369 (6)
C3—C16	1.527 (2)	C27—C32	1.386 (10)
C3—C4	1.539 (2)	C28—C29	1.390 (11)
C3—H3	0.9800	C28—H28	0.9300
C4—C5	1.536 (2)	C29—C30	1.374 (12)
C4—H4A	0.9700	C29—H29	0.9300
C4—H4B	0.9700	C30—C31	1.341 (5)
C5—O1	1.4453 (19)	C30—H30	0.9300
C5—C13	1.524 (2)	C31—C32	1.396 (8)
O1—C6	1.380 (2)	C31—H31	0.9300
C6—C7	1.372 (3)	C32—C33	1.448 (10)
C6—C11	1.403 (3)	C33—C34	1.341 (5)
C7—C8	1.401 (3)	C33—H33	0.9300
C7—H7	0.9300	C34—C35	1.463 (13)
C8—C9	1.360 (4)	C35—O6	1.203 (13)
C8—H8	0.9300	C35—O7	1.398 (13)
C9—C10	1.361 (4)	O7—C36	1.438 (5)
C9—H9	0.9300	C36—H36A	0.9600
C10—C11	1.405 (3)	C36—H36B	0.9600
C10—H10	0.9300	C36—H36C	0.9600
C11—C12	1.440 (3)	O5'—C27'	1.378 (4)
C12—C13	1.335 (2)	C27'—C28'	1.358 (5)
C12—H12	0.9300	C27'—C32'	1.379 (7)
C13—C14	1.481 (3)	C28'—C29'	1.391 (5)
C14—O2	1.203 (2)	C28'—H28'	0.9300
C14—O3	1.360 (2)	C29'—C30'	1.368 (6)
O3—C15	1.474 (3)	C29'—H29'	0.9300
C15—H15A	0.9600	C30'—C31'	1.345 (10)
C15—H15B	0.9600	C30'—H30'	0.9300
C15—H15C	0.9600	C31'—C32'	1.406 (10)
C16—C21	1.390 (2)	C31'—H31'	0.9300
C16—C17	1.391 (2)	C32'—C33'	1.467 (10)
C17—O4	1.375 (2)	C33'—C34'	1.354 (8)
C17—C18	1.394 (2)	C33'—H33'	0.9300
C18—C19	1.378 (2)	C34'—C35'	1.454 (6)
C18—H18	0.9300	C35'—O6'	1.202 (5)
C19—C20	1.388 (3)	C35'—O7'	1.384 (8)
C19—H19	0.9300	O7'—C36'	1.448 (8)
C20—C21	1.392 (2)	C36'—H36D	0.9600
C20—H20	0.9300	C36'—H36E	0.9600
C21—H21	0.9300	C36'—H36F	0.9600
O4—H4C	0.8200	C37—C42	1.386 (2)
N5—C23	1.3479 (19)	C37—C38	1.399 (2)
N5—N6	1.3931 (18)	C38—O8	1.362 (2)
N5—C26	1.411 (2)	C38—C39	1.395 (2)
N6—C22	1.308 (2)	C39—C40	1.373 (3)
C22—N7	1.364 (2)	C39—H39	0.9300
C22—H22	0.9300	C40—C41	1.371 (3)
N7—C23	1.326 (2)	C40—H40	0.9300
C23—N8	1.349 (2)	C41—C42	1.390 (3)
N8—C24	1.4581 (19)	C41—H41	0.9300
N8—H8A	0.8600	C42—H42	0.9300
C24—C37	1.519 (2)	O8—H8B	0.8200
C24—C25	1.527 (2)		
			
C2—N1—N2	109.09 (11)	C37—C24—H24	108.5
C2—N1—C5	126.44 (12)	C25—C24—H24	108.5
N2—N1—C5	123.44 (11)	C24—C25—C26	110.53 (14)
C1—N2—N1	101.84 (11)	C24—C25—H25A	109.5
N2—C1—N3	116.56 (13)	C26—C25—H25A	109.5
N2—C1—H1	121.7	C24—C25—H25B	109.5
N3—C1—H1	121.7	C26—C25—H25B	109.5
C2—N3—C1	102.10 (12)	H25A—C25—H25B	108.1
N3—C2—N4	128.49 (13)	O5—C26—N5	112.9 (2)
N3—C2—N1	110.40 (13)	O5—C26—C34'	76.8 (2)
N4—C2—N1	121.10 (13)	N5—C26—C34'	120.6 (2)
C2—N4—C3	116.58 (12)	O5—C26—C25	115.8 (2)
C2—N4—H4	121.7	N5—C26—C25	110.47 (13)
C3—N4—H4	121.7	C34'—C26—C25	116.5 (2)
N4—C3—C16	112.15 (12)	N5—C26—O5'	97.70 (18)
N4—C3—C4	107.46 (12)	C34'—C26—O5'	103.0 (2)
C16—C3—C4	110.81 (12)	C25—C26—O5'	104.97 (18)
N4—C3—H3	108.8	O5—C26—C34	109.8 (2)
C16—C3—H3	108.8	N5—C26—C34	103.2 (2)
C4—C3—H3	108.8	C25—C26—C34	103.4 (2)
C5—C4—C3	112.01 (12)	O5'—C26—C34	135.8 (2)
C5—C4—H4A	109.2	C26—O5—C27	121.2 (4)
C3—C4—H4A	109.2	C28—C27—C32	122.6 (5)
C5—C4—H4B	109.2	C28—C27—O5	117.1 (5)
C3—C4—H4B	109.2	C32—C27—O5	120.3 (5)
H4A—C4—H4B	107.9	C27—C28—C29	117.5 (7)
N1—C5—O1	103.95 (12)	C27—C28—H28	121.3
N1—C5—C13	111.11 (13)	C29—C28—H28	121.3
O1—C5—C13	110.71 (12)	C30—C29—C28	121.2 (9)
N1—C5—C4	108.48 (11)	C30—C29—H29	119.4
O1—C5—C4	109.72 (13)	C28—C29—H29	119.4
C13—C5—C4	112.50 (13)	C31—C30—C29	119.9 (6)
C6—O1—C5	117.69 (13)	C31—C30—H30	120.1
C7—C6—O1	118.12 (19)	C29—C30—H30	120.1
C7—C6—C11	121.48 (19)	C30—C31—C32	121.7 (5)
O1—C6—C11	120.33 (17)	C30—C31—H31	119.2
C6—C7—C8	118.7 (3)	C32—C31—H31	119.2
C6—C7—H7	120.6	C27—C32—C31	117.1 (7)
C8—C7—H7	120.6	C27—C32—C33	118.5 (6)
C9—C8—C7	120.4 (3)	C31—C32—C33	124.4 (7)
C9—C8—H8	119.8	C34—C33—C32	119.9 (5)
C7—C8—H8	119.8	C34—C33—H33	120.1
C8—C9—C10	121.3 (2)	C32—C33—H33	120.1
C8—C9—H9	119.4	C33—C34—C35	121.6 (8)
C10—C9—H9	119.4	C33—C34—C26	118.0 (4)
C9—C10—C11	120.4 (3)	C35—C34—C26	120.4 (6)
C9—C10—H10	119.8	O6—C35—O7	118.0 (11)
C11—C10—H10	119.8	O6—C35—C34	126.7 (12)
C6—C11—C10	117.8 (2)	O7—C35—C34	113.3 (9)
C6—C11—C12	117.85 (16)	C35—O7—C36	118.0 (6)
C10—C11—C12	124.4 (2)	C27'—O5'—C26	120.7 (3)
C13—C12—C11	120.83 (18)	C28'—C27'—O5'	118.8 (3)
C13—C12—H12	119.6	C28'—C27'—C32'	120.9 (4)
C11—C12—H12	119.6	O5'—C27'—C32'	120.1 (4)
C12—C13—C14	122.87 (17)	C27'—C28'—C29'	118.9 (4)
C12—C13—C5	120.33 (16)	C27'—C28'—H28'	120.5
C14—C13—C5	116.38 (14)	C29'—C28'—H28'	120.5
O2—C14—O3	123.40 (19)	C30'—C29'—C28'	120.8 (4)
O2—C14—C13	125.29 (17)	C30'—C29'—H29'	119.6
O3—C14—C13	111.25 (18)	C28'—C29'—H29'	119.6
C14—O3—C15	113.54 (19)	C31'—C30'—C29'	120.1 (6)
O3—C15—H15A	109.5	C31'—C30'—H30'	119.9
O3—C15—H15B	109.5	C29'—C30'—H30'	119.9
H15A—C15—H15B	109.5	C30'—C31'—C32'	120.2 (9)
O3—C15—H15C	109.5	C30'—C31'—H31'	119.9
H15A—C15—H15C	109.5	C32'—C31'—H31'	119.9
H15B—C15—H15C	109.5	C27'—C32'—C31'	118.9 (7)
C21—C16—C17	119.01 (15)	C27'—C32'—C33'	118.1 (5)
C21—C16—C3	122.38 (14)	C31'—C32'—C33'	123.0 (7)
C17—C16—C3	118.57 (14)	C34'—C33'—C32'	120.5 (7)
O4—C17—C16	117.39 (14)	C34'—C33'—H33'	119.8
O4—C17—C18	122.45 (15)	C32'—C33'—H33'	119.8
C16—C17—C18	120.16 (16)	C33'—C34'—C35'	118.3 (5)
C19—C18—C17	120.05 (16)	C33'—C34'—C26	125.5 (5)
C19—C18—H18	120.0	C35'—C34'—C26	115.1 (3)
C17—C18—H18	120.0	O6'—C35'—O7'	118.6 (5)
C18—C19—C20	120.66 (17)	O6'—C35'—C34'	126.0 (4)
C18—C19—H19	119.7	O7'—C35'—C34'	115.3 (5)
C20—C19—H19	119.7	C35'—O7'—C36'	118.8 (6)
C19—C20—C21	118.98 (17)	O7'—C36'—H36D	109.5
C19—C20—H20	120.5	O7'—C36'—H36E	109.5
C21—C20—H20	120.5	H36D—C36'—H36E	109.5
C16—C21—C20	121.12 (17)	O7'—C36'—H36F	109.5
C16—C21—H21	119.4	H36D—C36'—H36F	109.5
C20—C21—H21	119.4	H36E—C36'—H36F	109.5
C17—O4—H4C	109.5	C42—C37—C38	118.92 (15)
C23—N5—N6	108.78 (12)	C42—C37—C24	122.97 (14)
C23—N5—C26	125.94 (14)	C38—C37—C24	118.11 (14)
N6—N5—C26	125.16 (12)	O8—C38—C39	122.79 (15)
C22—N6—N5	101.97 (13)	O8—C38—C37	117.45 (15)
N6—C22—N7	116.03 (15)	C39—C38—C37	119.76 (16)
N6—C22—H22	122.0	C40—C39—C38	119.85 (17)
N7—C22—H22	122.0	C40—C39—H39	120.1
C23—N7—C22	102.52 (13)	C38—C39—H39	120.1
N7—C23—N5	110.68 (14)	C41—C40—C39	121.29 (18)
N7—C23—N8	128.47 (14)	C41—C40—H40	119.4
N5—C23—N8	120.85 (14)	C39—C40—H40	119.4
C23—N8—C24	117.21 (13)	C40—C41—C42	119.12 (19)
C23—N8—H8A	121.4	C40—C41—H41	120.4
C24—N8—H8A	121.4	C42—C41—H41	120.4
N8—C24—C37	112.18 (13)	C37—C42—C41	121.03 (18)
N8—C24—C25	107.67 (13)	C37—C42—H42	119.5
C37—C24—C25	111.29 (13)	C41—C42—H42	119.5
N8—C24—H24	108.5	C38—O8—H8B	109.5
			
C2—N1—N2—C1	−0.15 (15)	N6—N5—C26—C34	−56.8 (3)
C5—N1—N2—C1	−169.24 (13)	C24—C25—C26—O5	89.4 (2)
N1—N2—C1—N3	0.28 (17)	C24—C25—C26—N5	−40.6 (2)
N2—C1—N3—C2	−0.29 (18)	C24—C25—C26—C34'	177.0 (2)
C1—N3—C2—N4	−179.00 (15)	C24—C25—C26—O5'	63.80 (19)
C1—N3—C2—N1	0.17 (16)	C24—C25—C26—C34	−150.4 (2)
N2—N1—C2—N3	−0.01 (16)	N5—C26—O5—C27	−156.1 (4)
C5—N1—C2—N3	168.67 (13)	C34'—C26—O5—C27	−38.2 (4)
N2—N1—C2—N4	179.22 (13)	C25—C26—O5—C27	75.1 (4)
C5—N1—C2—N4	−12.1 (2)	O5'—C26—O5—C27	145.8 (8)
N3—C2—N4—C3	165.60 (15)	C34—C26—O5—C27	−41.5 (5)
N1—C2—N4—C3	−13.5 (2)	C26—O5—C27—C28	−149.3 (4)
C2—N4—C3—C16	169.72 (13)	C26—O5—C27—C32	32.6 (8)
C2—N4—C3—C4	47.70 (17)	C32—C27—C28—C29	1.2 (14)
N4—C3—C4—C5	−60.47 (16)	O5—C27—C28—C29	−176.8 (12)
C16—C3—C4—C5	176.68 (13)	C27—C28—C29—C30	1(2)
C2—N1—C5—O1	114.85 (15)	C28—C29—C30—C31	−1(2)
N2—N1—C5—O1	−77.99 (16)	C29—C30—C31—C32	−0.9 (13)
C2—N1—C5—C13	−126.03 (16)	C28—C27—C32—C31	−2.7 (12)
N2—N1—C5—C13	41.12 (18)	O5—C27—C32—C31	175.3 (6)
C2—N1—C5—C4	−1.9 (2)	C28—C27—C32—C33	−180.0 (6)
N2—N1—C5—C4	165.28 (13)	O5—C27—C32—C33	−2.0 (11)
C3—C4—C5—N1	37.65 (17)	C30—C31—C32—C27	2.5 (11)
C3—C4—C5—O1	−75.30 (15)	C30—C31—C32—C33	179.6 (6)
C3—C4—C5—C13	160.98 (13)	C27—C32—C33—C34	−11.3 (11)
N1—C5—O1—C6	160.34 (14)	C31—C32—C33—C34	171.6 (7)
C13—C5—O1—C6	40.94 (19)	C32—C33—C34—C35	−178 (2)
C4—C5—O1—C6	−83.81 (17)	C32—C33—C34—C26	−0.6 (7)
C5—O1—C6—C7	151.29 (18)	O5—C26—C34—C33	26.3 (5)
C5—O1—C6—C11	−31.8 (2)	N5—C26—C34—C33	147.0 (3)
O1—C6—C7—C8	177.3 (2)	C34'—C26—C34—C33	20.4 (3)
C11—C6—C7—C8	0.4 (3)	C25—C26—C34—C33	−97.9 (4)
C6—C7—C8—C9	−0.7 (4)	O5'—C26—C34—C33	31.0 (5)
C7—C8—C9—C10	0.7 (5)	O5—C26—C34—C35	−156 (2)
C8—C9—C10—C11	−0.5 (4)	N5—C26—C34—C35	−36 (2)
C7—C6—C11—C10	−0.1 (3)	C34'—C26—C34—C35	−162 (2)
O1—C6—C11—C10	−176.96 (18)	C25—C26—C34—C35	79 (2)
C7—C6—C11—C12	−178.9 (2)	O5'—C26—C34—C35	−152 (2)
O1—C6—C11—C12	4.2 (3)	C33—C34—C35—O6	155 (3)
C9—C10—C11—C6	0.1 (3)	C26—C34—C35—O6	−22 (5)
C9—C10—C11—C12	178.9 (2)	C33—C34—C35—O7	−8(4)
C6—C11—C12—C13	10.9 (3)	C26—C34—C35—O7	174.6 (17)
C10—C11—C12—C13	−167.8 (2)	O6—C35—O7—C36	4(4)
C11—C12—C13—C14	173.58 (17)	C34—C35—O7—C36	169.2 (17)
C11—C12—C13—C5	1.4 (3)	O5—C26—O5'—C27'	−36.4 (5)
N1—C5—C13—C12	−141.31 (16)	N5—C26—O5'—C27'	−164.3 (3)
O1—C5—C13—C12	−26.3 (2)	C34'—C26—O5'—C27'	−40.4 (3)
C4—C5—C13—C12	96.84 (18)	C25—C26—O5'—C27'	82.0 (3)
N1—C5—C13—C14	45.98 (19)	C34—C26—O5'—C27'	−46.3 (5)
O1—C5—C13—C14	160.96 (14)	C26—O5'—C27'—C28'	−152.5 (3)
C4—C5—C13—C14	−75.87 (18)	C26—O5'—C27'—C32'	32.1 (5)
C12—C13—C14—O2	−161.1 (2)	O5'—C27'—C28'—C29'	−178.2 (4)
C5—C13—C14—O2	11.4 (3)	C32'—C27'—C28'—C29'	−2.7 (6)
C12—C13—C14—O3	21.6 (3)	C27'—C28'—C29'—C30'	0.6 (6)
C5—C13—C14—O3	−165.94 (16)	C28'—C29'—C30'—C31'	2.1 (13)
O2—C14—O3—C15	6.1 (3)	C29'—C30'—C31'—C32'	−3(2)
C13—C14—O3—C15	−176.5 (2)	C28'—C27'—C32'—C31'	2.1 (12)
N4—C3—C16—C21	−24.5 (2)	O5'—C27'—C32'—C31'	177.5 (11)
C4—C3—C16—C21	95.55 (18)	C28'—C27'—C32'—C33'	179.0 (5)
N4—C3—C16—C17	157.78 (14)	O5'—C27'—C32'—C33'	−5.6 (7)
C4—C3—C16—C17	−82.13 (17)	C30'—C31'—C32'—C27'	1(2)
C21—C16—C17—O4	−179.75 (15)	C30'—C31'—C32'—C33'	−176.1 (12)
C3—C16—C17—O4	−2.0 (2)	C27'—C32'—C33'—C34'	−7.0 (9)
C21—C16—C17—C18	0.0 (2)	C31'—C32'—C33'—C34'	169.7 (12)
C3—C16—C17—C18	177.78 (15)	C32'—C33'—C34'—C35'	−175.5 (5)
O4—C17—C18—C19	178.58 (17)	C32'—C33'—C34'—C26	−8.1 (9)
C16—C17—C18—C19	−1.2 (3)	O5—C26—C34'—C33'	26.7 (5)
C17—C18—C19—C20	1.5 (3)	N5—C26—C34'—C33'	135.8 (5)
C18—C19—C20—C21	−0.7 (3)	C25—C26—C34'—C33'	−85.8 (5)
C17—C16—C21—C20	0.8 (3)	O5'—C26—C34'—C33'	28.5 (5)
C3—C16—C21—C20	−176.86 (16)	C34—C26—C34'—C33'	−159.0 (7)
C19—C20—C21—C16	−0.5 (3)	O5—C26—C34'—C35'	−165.5 (3)
C23—N5—N6—C22	−0.57 (19)	N5—C26—C34'—C35'	−56.5 (4)
C26—N5—N6—C22	175.70 (17)	C25—C26—C34'—C35'	82.0 (3)
N5—N6—C22—N7	−0.3 (2)	O5'—C26—C34'—C35'	−163.7 (3)
N6—C22—N7—C23	1.1 (2)	C34—C26—C34'—C35'	8.7 (4)
C22—N7—C23—N5	−1.4 (2)	C33'—C34'—C35'—O6'	−16.3 (7)
C22—N7—C23—N8	178.95 (19)	C26—C34'—C35'—O6'	175.0 (4)
N6—N5—C23—N7	1.3 (2)	C33'—C34'—C35'—O7'	161.1 (11)
C26—N5—C23—N7	−174.92 (17)	C26—C34'—C35'—O7'	−7.6 (11)
N6—N5—C23—N8	−179.02 (16)	O6'—C35'—O7'—C36'	1(2)
C26—N5—C23—N8	4.7 (3)	C34'—C35'—O7'—C36'	−176.6 (11)
N7—C23—N8—C24	−163.94 (18)	N8—C24—C37—C42	23.0 (2)
N5—C23—N8—C24	16.5 (2)	C25—C24—C37—C42	−97.74 (18)
C23—N8—C24—C37	−170.38 (15)	N8—C24—C37—C38	−157.29 (14)
C23—N8—C24—C25	−47.6 (2)	C25—C24—C37—C38	82.00 (18)
N8—C24—C25—C26	59.30 (19)	C42—C37—C38—O8	177.87 (15)
C37—C24—C25—C26	−177.38 (14)	C24—C37—C38—O8	−1.9 (2)
C23—N5—C26—O5	−122.6 (2)	C42—C37—C38—C39	−1.6 (2)
N6—N5—C26—O5	61.7 (3)	C24—C37—C38—C39	178.66 (14)
C23—N5—C26—C34'	149.5 (2)	O8—C38—C39—C40	−178.60 (16)
N6—N5—C26—C34'	−26.1 (3)	C37—C38—C39—C40	0.8 (3)
C23—N5—C26—C25	8.9 (3)	C38—C39—C40—C41	−0.6 (3)
N6—N5—C26—C25	−166.78 (15)	C39—C40—C41—C42	1.1 (3)
C23—N5—C26—O5'	−100.3 (2)	C38—C37—C42—C41	2.1 (3)
N6—N5—C26—O5'	84.0 (2)	C24—C37—C42—C41	−178.11 (16)
C23—N5—C26—C34	118.9 (2)	C40—C41—C42—C37	−1.9 (3)

### Hydrogen-bond geometry (Å, °)

**Table:**

*D*—H···*A*	*D*—H	H···*A*	*D*···*A*	*D*—H···*A*
N4—H4···N7	0.86	2.21	3.0156 (19)	156.
O4—H4C···N2^i^	0.82	2.12	2.9152 (17)	163.
N8—H8A···N3	0.86	2.10	2.9168 (19)	159.
O8—H8B···N6^ii^	0.82	2.09	2.8898 (19)	164.

Symmetry codes: (i) −*x*+2, *y*+1/2, −*z*+1/2; (ii) −*x*+1, *y*−1/2, −*z*+1/2.
